# Bibliographical Notices

**Published:** 1848-08

**Authors:** 


### EDITORIAL DEPARTMENT.

### BIBLIOGRAPHICAL NOTICES.

*On the Theory and Practice of Midwifery.* By Fleetwood Churchill, M. D., M. R. I. A., etc., with notes and additions, by Robert M. Huston, M. D., Professor of Materia Medica and General Therapeutics, and formerly of Obstetrics, and the Diseases of Women and Children in the Jefferson Medical College of Philadelphia, etc., etc. Third American Edition, Revised and improved by the author. With one hundred and twenty-eight illustrations from drawings by Bogg and others. Engraved by Gilbert. Philadelphia: Lee and Blanchard. 1848. 8vo. pp. 525.

The first American edition of this work was published in 1843. In 1845 a second edition was called for. The latter having been exhausted, a third edition is now issued, to meet an increasing demand for the work. These facts speak for the excellence of the work, far better than any commendations from the pen of the Journalist. It suffices to chronicle a new edition, which, we learn from the preface, has undergone a careful revisal by both author and editor, new matter also being added to present to the reader the new facts and observations of interest and importance that have transpired since the last edition was issued.

---

*On the Blood and Urine.* By John William Griffith, M. D., F. L. S., &c., G. Owen Rees, M. D., F. R. S., F. G. S., &c., and Alfred Markwick, M. D., &c. In one volume; Philadelphia, Lea & Blanchard, 1848. 12 mo.

This volume embraces three distinct works, which were published in London separately. The subjects treated, being closely connected, and each of the works being too small to make a book of much size, the American publishers, very judiciously, as we think, have embodied them in a single volume, the individuality of each work, however, being preserved.

The work by Dr. Griffith is entitled "A practical manual, containing a description of the general, chemical, and microscopical characters of the Blood, and secretions of the human body, as well as their components, including both their healthy and diseased states; with the best methods of separating and estimating their ingredients; also, a succinct account of the various concretions occasionally found in the body, and forming calculi." This work occupies 182 pages.

The title-page of the work by Dr. Rees, reads thus:—" On the analysis of the Blood and Urine, in health and disease; and on the treatment of urinary diseases." This work embraces 165 pages.

Dr. Markwick's treatise is "A guide to the examination of the urine in health and disease, for the use of students." 113 pages.

Students and Practitioners who would not be laggard in following the march of practical Medicine, must not neglect the subjects treated of in these three *brochures*

---

*A Dispensatory and Therapeutical Remembrancer;* comprising the entire lists of Materia Medica, preparations and compounds, with a full and distinct version of every Practical Formula, as authorized by the London, Edinburgh and Dublin Royal College of physicians, in the latest editions of their several *Pharmacopœias;* to which are subjoined *copious relative tables,* exemplifying approved forms under which compatible medicines, &c., may be extemporaneously combined, &c., &c., &c. By John Mayne, M. D., L. R. C. S., Edinburgh. Revised, with the addition of the Formulæ of the *United States Pharmacopœias,* etc. By. R. Eglesfield Griffith, M. D., author of "Medical Botany," etc., etc. Philadelphia: Lea and Blanchard. 1838. 12mo. pp. 329.

The title page of this work, which, it will be perceived, is unusually copious, sufficiently explains its character and scope. It is truly a *multum in parvo,* and must be a convenient volume for reference.

---

*A Practical Treatise on the Diseases of Children.* By. J. Forsyth Meigs, M. D., Lecturer on the Diseases of Children, in the Philadelphia Medical Association, &c. Philadelphia: Lindsay & Blackiston. 1848. 8vo. pp. 575.

This volume is No. 3, of the *Medical Practitioners' and Students' Library,* the two first volumes of the series having been already noticed in our pages. We have given the greater part of the work a careful perusal, and, in our opinion, it is a production in every way creditable to the Author. Were we disposed to be critical, we might comment on the very constant reference to the author's experience, which, if we mistake not, has extended over a less number of years than that of some other authors of practical treatises on the diseases of children. We do not intend by this remark to sneer at the crime of being a young man; we would only intimate that the manner in which the author speaks of his own experience, may, possibly, convey an erroneous impression, appearing to imply the possession of some gray hairs. That the author has prosecuted personal observation in a careful, philosophical manner, and turned his opportunities to the best account, the work bears evidence. His arrangement of topics, and the practical conclusions at which he arrives, appear to us to be sound and judicious. Moreover the work embodies, in a small compass, the most valuable precepts of the most approved modern authorities, on the subjects of which it treats. In short, it is a clearly written book, of which the author need not be ashamed hereafter, even if he achieve still more creditable bibliographical efforts.

It is published to correspond with the numbers of the same series already issued, and may be sent by mail, the price being $1 25.

The project of the *Library*, and the "getting up" of the works are creditable to the enterprising publishers, Messrs. Lindsay & Blakiston.

---

*A Case of Typhus or Ship Fever, with remarks.* By WM. INGALLS, M. D., Fellow of the Massachusetts, Rhode Island and New Hampshire Medical Societies; formerly Professor of Anatomy, Surgery, and Physiology in Brown University.

Our organ of reverence leads us to shrink from any apparent want of respectful consideration for one, whose advanced age, and former position as a practitioner, are well known to us. We therefore leave this little pamphlet without criticism.

